# Evaluation of Serum miR-17-92 Cluster as Noninvasive Biomarkers for Bladder Cancer Diagnosis

**DOI:** 10.3389/fonc.2021.795837

**Published:** 2021-12-22

**Authors:** Jingyao Wang, Xiqi Peng, Rongkang Li, Kaihao Liu, Chunduo Zhang, Xuan Chen, Guocheng Huang, Liwen Zhao, Zebo Chen, Yongqing Lai

**Affiliations:** ^1^ Department of Urology, Guangdong and Shenzhen Key Laboratory of Male Reproductive Medicine and Genetics, Peking University Shenzhen Hospital, Shenzhen, China; ^2^ Shantou University Medical College, Shantou, China; ^3^ Anhui Medical University, Hefei, China; ^4^ Department of Urology, Peking University Shenzhen Hospital, Shenzhen, China

**Keywords:** bladder cancer, circulating biomarker, non-invasive detection, diagnosis, logistic regression model

## Abstract

Previous studies have shown that the miR-17-92 cluster is involved in the occurrence and development of bladder cancer. However, the role of serum miR-17-92 cluster in the diagnosis of bladder cancer has not been studied. In the present study, we evaluated the expression of miR-17-92 cluster members in bladder cancer tissues by analyzing 428 cases from TCGA database. Next, we collected the sera of 74 bladder cancer patients and 90 controls, and used qRT-PCR to detect the relative expression of the cluster. The results showed that the expression of the cluster members in the sera of patients were significantly higher than that of the controls, and they were positively correlated with the clinical stage and pathological grade of the patients. We evaluated their ability to diagnose bladder cancer using ROC, of which miR-92a-3p (AUC = 0.902), miR-17-5p (AUC = 0.845) and miR-20a-5p (AUC = 0.806) were the most prominent. Finally, we established a diagnostic model by logistic regression (AUC = 0.969). We further validated the results of the study using another dataset from the GEO database. Moreover, we evaluated the prognostic value of the cluster. The results revealed that miR-20a-5p was correlated with recurrence of bladder cancer. In summary, the present study validated the overexpression of serum miR-17-92 cluster in bladder cancer. The model composed of the three cluster members were confirmed to be a promising noninvasive biomarker for bladder cancer diagnosis.

## Introduction

Bladder cancer (BC) is the sixth most common cancer worldwide, with the eighth highest mortality rate (1). Unfortunately, little improvement in the diagnosis and treatment of BC has been made over the past three decades, unlike many other tumors (2). The main reason was that the existing diagnostic methods for BC cannot meet the needs of clinical work. A good BC diagnosis method can not only help the early diagnosis and treatment of BC, but can also monitor the recurrence after surgery. At present, cystoscopy is the gold standard for detecting BC, but its detection method is invasive (3). It is usually accompanied by risks of bleeding, UTI, and difficulty urinating, so it is not suitable for early cancer screening. Also, long-term frequent cystoscopy after surgery will bring a great mental burden to patients (4). Therefore, it is necessary to find new feasible diagnostic methods for BC.

In recent years, the research on BC diagnostic biomarkers has aroused great interest, but most of them has not yet been clinically available (5, 6). Biomarkers with high specificity and sensitivity from body fluids may be effective tools for non-invasive BC detection.

microRNAs (miRNAs) are small non-coding and endogenous RNAs which can bind to the 3′-UTR of target mRNAs, regulate gene expression and thus lead to mRNA degradation or translation inhibition (7, 8). Mounting evidence has shown that circulating miRNAs are promising biomarkers for tumor detection for the reason that miRNAs can be stably detected in circulating blood (9–11).

Many miRNAs are located in polycistronic miRNA “clusters”, where multiple miRNAs are produced from a single primary transcript. The miR-17-92 cluster, also named as oncomiR-1, is a frequently amplified locus in cancer (12). The cluster encodes six mature miRNAs: miR-17-5p, miR-18a-5p, miR-19a-3p, miR-20a-5p, miR-19b-3p and miR-92a-3p. Many studies have reported that members of the miR-17-92 cluster are upregulated in BC cells and play a carcinogenic role (13–17). Therefore, we want to know whether this cluster can be used as new circulating markers to diagnose BC.

In the present study, we tested the relative expression levels of miR-17-92 cluster members in BC tissues and patient sera. We evaluated the diagnostic ability of serum miR-17-92 cluster. We also constructed a three-miRNA model, which has a high sensitivity and specificity for BC diagnosis. These results were also confirmed in the external verification set. Furtherly, we explored the relationship between the cluster and BC recurrence.

## Materials and Methods

### Study Design

**Figure 1:**
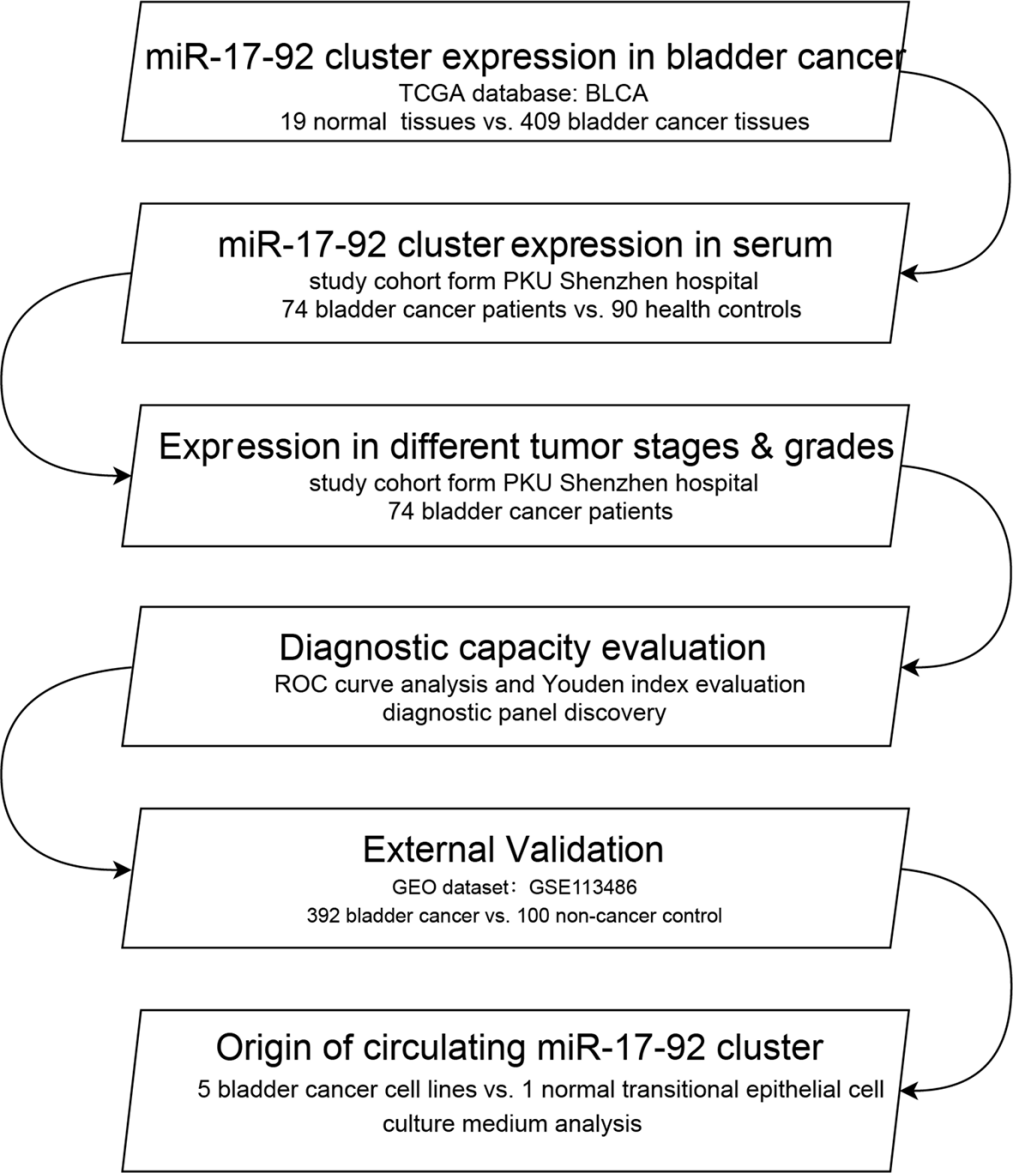
The flowchart of the study design. BLCA, bladder urothelial carcinoma; ROC, receiver operating characteristic.

We firstly tested the differential expression of the miR-17-92 cluster (miR-17-5p, miR-18a-5p, miR-19a-3p, miR-20a-5p, miR-19b-3p and miR-92a-3p) in BC and adjacent normal bladder tissues using data from The Cancer Genome Atlas (TCGA) database. We then analyzed the differential expression of circulating miR-17-92 cluster in the sera from 74 BC patients and 90 healthy controls (HCs) using the quantitative reverse transcription-polymerase chain reaction (qRT-PCR) method. Also, we discovered the expression trends of miR-17-92 cluster members of BC patients with different pathological grades and stages. We evaluated the diagnostic ability of miR-17-92 cluster by ROC analysis. A logistic regression model was established to enhance the sensitivity and specificity of diagnosis. A GEO dataset (including 492 samples) was used as an external validation set to furtherly confirmed the diagnostic value of serum miR-17-92 cluster in BC. We also test the expression of the cluster in BC cell lines and a normal transitional epithelial cell line (SV-HUC-1). The culture medium was used to illustrate the origin of circulating miRNAs. Moreover, Cox regression analysis and Kaplan-Meier analysis were used to evaluate their relationship with BC recurrence. The design of this study was shown in **Figure 1**.

### Enrollment of Participants

The present study enrolled a total of 74 BC patients from Peking University Shenzhen Hospital between June 2017 and July 2019. All serum samples were collected before accepting any treatment. The histological grade was classified according to the standards of World Health Organization. The tumor stage was confirmed based the TNM staging system. We enrolled a total of 90 HCs who came to the hospital for physical examination and had no history to tumors and other diseases. The age and gender of HCs and BC patients were matched. The demographic and clinical characteristics of the participants was listed in **Table 1**. No significant differences were found in age or gender distribution among BC and HCs group (p > 0.05). All participants in this study had signed informed consent forms before their blood samples being collected, and agreed to be included in this study. This study had been approved by the Ethics Committee of Peking University Shenzhen Hospital. The study was processed following the Declaration of Helsinki. The specimen collection process was implemented according to regulations of the committee.

**Table 1:** Demographic and clinical characteristics of 164 participants enrolled in the study.

	BC patients	HCs
**Total number**	74	90
**Age** (Mean ± SD)	63.8 ± 13.5	56.6 ± 13.1
**Gender** (%)		
Male	54 (73.0)	42 (46.7)
Female	20 (27.0)	48 (53.3)
**Tumor stage** (%)		
TaN0M0	26 (35.1)	
T1N0M0	30 (40.5)	
T2N0M0	13 (17.6)	
≥pT3	5 (6.8)	
**Pathological grade** (%)		
Low grade	35 (47.3)	
High grade	39 (52.7)	

BC, bladder cancer; HCs, healthy controls.

### Data Acquisition

We obtained the miRNA-sequencing data of the Bladder Urothelial Carcinoma (BLCA) from TCGA database, which contained 19 normal bladder epithelial tissues, 409 bladder urothelial carcinoma tissues. The corresponding clinical information of the patients were also obtained. A serum miRNA expression profile matrix GSE113486, which consist of serum samples from multiple tumors and normal controls, were downloaded from the GEO database. It was performed on GPL21263 Toray Industries platform. The expression data and clinical information of 392 BC patients and 100 non-cancer controls inside it were utilized in our study. All miRNA expression data were standardized and log2 transformation for further analysis.

### Serum Sample Collection

We collected 10ml peripheral blood of each participant before their accepting any treatment. The peripheral blood was centrifuged at 1000 g for 10 minutes and 15,000 g for 5 minutes at 4°C within 2 hours. 2 µl of 10 nmol/L synthetic C. Elegans miRNA-39 (RiboBio, Guangzhou, China) was spiked into each serum sample before the experiments to control the variability during the extraction and purification process.

### Cell Culture

The normal transitional epithelial cell (SV-HUC-1) and BC cell lines including RT4, J82, UM-UC-3, 5637 and T24, were obtained from Shanghai Institute of Biochemistry and Cell Biology (Shanghai, China). The cells were maintained in Dulbecco’s modified Eagle’s medium (DMEM; Gibco; Thermo Fisher Scientific. Inc., Waltham, MA, USA) or RPMI-1640 (Gibco; Thermo Fisher Scientific, Inc., Waltham, MA, USA) supplemented with fetal bovine serum (FBS; 10% Gibco; Thermo Fisher Scientific, Inc.), antibiotics (1% 100 μl/ml penicillin and 100 mg/ml streptomycin sulfates) and glutamine (1%) in 37°C with 5% CO2. We collected the culture media from culture plates after the cells were cultivated for 24h, 48h, and 72h.

### RNA Extraction and Quantitative Reverse Transcription-Polymerase Chain Reaction

Total RNA was extracted from sera, cell lines and culture media using the TRIzol LS isolation kit (Thermo Fisher Scientific, Waltham, MA, USA) in accordance with the manufacturer’s instructions. Later, total RNA was resuspended with 30 µl RNase-free water and stored at −80°C for further experiments. Using the NanoDrop 2000 spectrophotometer (NanoDrop, Wilmington, DE, USA), we evaluated the concentration and purity of the extracted RNA.

The amplification of miRNAs was conducted using the specific reverse transcription primers from Bulge-Loop miRNA qRT-PCR Primer Set (RiboBio, Guangzhou, China). The real-time polymerase chain reaction was performed using SYBR Green qPCR kit (SYBR Pre-mix Ex Taq II, TaKaRa) in 384-well plates on LightCycler 480 Real-Time PCR System (Roche Diagnostics, Mannheim, Germany) at 95°C for 30 s, followed by 35 cycles in 95°C for 10 s, 60°C for 20 s and then 70°C for 10 s. The specificity of the PCR product was confirmed by melting curve analysis at last. The relative expression levels of target miRNAs were calculated by the 2−△△Cq method (18) and normalized to the spiked-in control cel-miR-39. All reactions had repeated three times or more.

### Statistical Analysis

The differential expression of each miRNA between BC and HCs groups were analyzed using Students’ T-test or Mann–Whitney U test. Multiple comparisons among different phases were analyzed using the Kruskal-Wallis rank test. Binary logistic regression analysis was performed to build the miRNA signature. And we evaluated the calibration by the Hosmer-Lemeshow goodness-of-fit test. Thee diagnostic ability of the miRNAs were evaluated by Receiver operating characteristic (ROC) curves and the area under the ROC curve (AUC). The optimal cut-off was determined by the Youden index (calculated as J = Sensitivity + Specificity − 1). Cox regression analysis and Kaplan-Meier analysis were used for prognostic analysis. We evaluated the calibration using the Hosmer-Lemeshow goodness-of-fit test. We used Receiver operating characteristic (ROC) curves and the area under the ROC curve (AUC) to evaluate the diagnostic ability of miRNAs. The Youden index was used to determine the optimal cut-off (Youden index= Sensitivity + Specificity − 1). Cox regression analysis and Kaplan-Meier analysis were used for prognostic analysis. All statistical analyses in this study were performed using SPSS software (Version 20, Chicago, USA), GraphPad Prism (Version 8, LaJolla, CA), and Medcalc (Version 19, Ostend, Belgium). Differences were considered to be significant when p-value was less than 0.05.

## Results

### Expression of miR-17-92 Cluster Members Was Remarkably Elevated in BC

**Figure 2:**
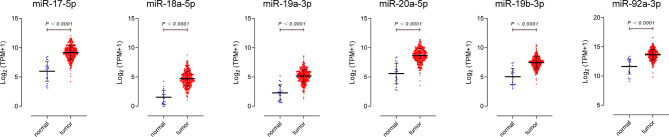
Expression profiles of miR-17-92 cluster members in bladder cancer tissues. Data were downloaded from The Cancer Genome Atlas database, including 19 para-carcinoma tissues (normal), 409 bladder urothelial carcinoma tissues(tumor).

We downloaded miRNA-isoform sequencing data of BLCA from the TCGA database, including 409 bladder cancer tissues and 19 normal bladder epithelium tissues. The differential expression ratio (logFC) and expression level (logTPM) of miR-17-92 cluster in BC tissues and para-carcinoma tissues are shown in **Table 2**. The higher the fold change (FC) value indicates the greater the difference in expression of the miRNA between the two groups, and it can better distinguish the two groups of samples. The higher the TPM value indicates the higher the expression level of the miRNA, which means it can be found in a smaller amount of sample. As shown in **Figure 2**, all 6 members of the miR-17-92 cluster are highly upregulated in BC tissues. The TPM value of miR-92a-3p is the highest among all. Such results support previous studies that the miR-17-92 cluster may play a carcinogenic role in BC.

**Table 2:** The expression profiles of miR-17-92 cluster members in BC tissues and patient sera.

miRNAs	Tissue (TCGA-BLCA)			Serum (PKU-shenzhen hospital)			Serum (GSE113486)		
	logFC	FDR	logTPM	logFC	p-value	AveCT	logFC	FDR	AveExpr
miR-17-5p	2.07	<0.01	9.37	1.81	<0.01	17.34	2.30	<0.01	2.02
miR-18a-5p	2.55	<0.01	4.97	1.34	0.03	20.10	2.64	<0.01	1.41
miR-19a-3p	1.93	<0.01	5.38	1.33	0.02	21.09	3.14	<0.01	0.56
miR-20a-5p	1.92	<0.01	8.89	1.93	<0.01	19.57	2.56	<0.01	1.45
miR-19b-3p	1.28	<0.01	7.57	1.71	<0.01	17.89	2.60	<0.01	2.26
miR-92a-3p	0.98	<0.01	13.72	1.89	<0.01	16.11	2.18	<0.01	6.32

FC, fold change; FDR, false discovery rate; TPM, Transcripts Per Kilobase of exonmodel per Million mapped reads; AveCT, average CT value; AveExpr, average expression.

**Figure 3:**
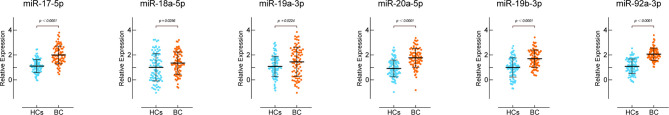
Expression of miR-17-92 cluster members in the sera of bladder cancer patients. HCs group includes sera of 90 healthy controls and BC group includes sera of 74 patients.

We detected the miR-17-92 cluster relative expression levels of the preoperative sera of 74 BC patients and the sera of 90 HCs. The results were consistent with tissue. As shown in **Figure 3** and **Table 2**, all members in miR-17-92 cluster were enriched in sera of BC patients. Among them, the expression differences of miR-18a-5p and miR-19a-3p in the two groups of sera were relatively small (0.05>P> 0.01), while the other miR-17-92 cluster members were significantly enriched in the sera of BC patients (P<0.01).

### Expression of miR-17-92 Cluster Members in Sera of Patients With Different Stages and Grades

**Figure 4:**
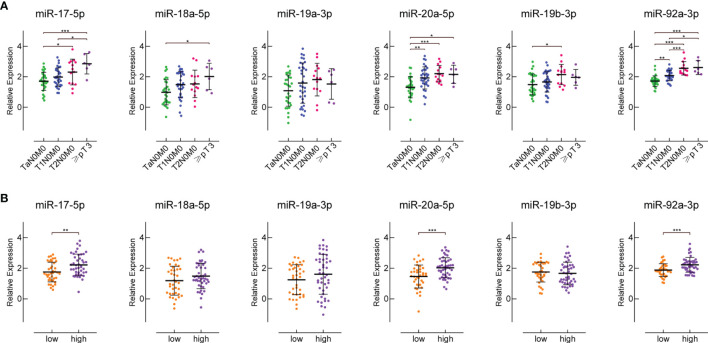
The expression of miR-17-92 cluster members in the sera of patients with different clinical stages and pathological grades. **(A)** shows their expression in the sera of patients with different clinical stages. There are 26 TaN0M0 patients, 30 T1N0M0 patients, 13 T2N0M0 patients, and 5 patients with ≥pT3. **(B)** shows their expression in the sera of patients with different pathological grades. There are 35 low-grade and 39 high-grade BC patients. *P < 0.05, **P < 0.01, ***P < 0.001.

We divided the 74 BC patients from our cohort into four groups based on different clinical stages, and into two groups based on different pathological grades. There were 5 patients with ≥pT3, 13 T2N0M0 patients, 30 T1N0M0 patients, and 26 TaN0M0 patients. There were 39 high-grade and 35 low-grade BC patients. The expression levels of miR-17-5p, miR-18a-5p, miR-20a-5p and miR-92a-3p in patients’ sera were significantly positively correlated with patients’ clinical grade (**Figure 4A**). But for miR-19a-3p and miR-19b-3p, this trend is not obvious. The expression levels of miR-17-5p, miR-20a-5p and miR-92a-3p in the sera of high-grade BC patients are much higher than those in low-grade patients (**Figure 4B**). The expression of serum miR-18a-5p, miR-19a-3p and miR-19b-3p was not related to BC pathological grade.

### Evaluation of the Diagnostic Ability of miR-17-92 Cluster Members in BC

**Figure 5:**
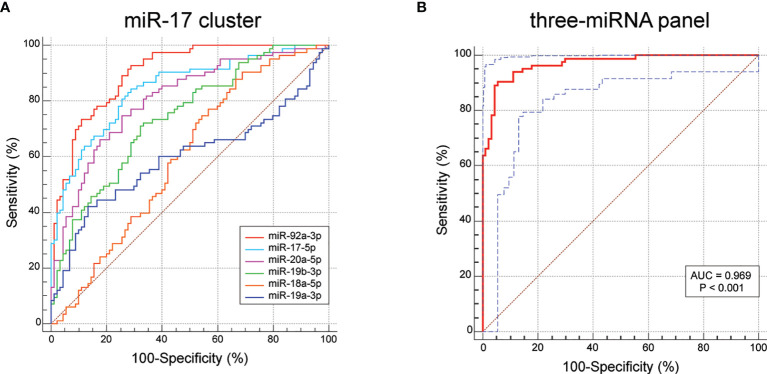
ROC results for the miR-17-92 cluster members and the three-miRNA diagnostic model (miR-130a-3p, miR-130b-3p and miR-301a-3p). **(A)** The AUC value of miR-17-5p is 0.845, miR-18a-5p is 0.597, miR-19a-3p is 0.596, miR-20a-5p is 0.806, miR-19b-3p is 0.741, miR-92a-3p is 0.902. **(B)** The AUC value of the three-miRNA model is 0.969 (95% CI: 0.931 - 0.989; sensitivity = 90.36%, specificity = 94.44%).

To assess the diagnostic value of the serum miR-17-92 cluster members in discriminating BC patients from healthy controls, ROC curve analyses were conducted. As shown in **Figure 5A** and **Table 3**, of the six miR-17-92 cluster members investigated, serum miR-92a-3p exhibited the highest accuracy in diagnosing BC, with an AUC of 0.902 (p = 0.022). Serum miR-17-5p (AUC = 0.845), miR-20a-5p (AUC = 0.806) and miR-19b-3p (AUC = 0.741) have moderate diagnostic ability. In contrast, serum miR-18a-5p (AUC = 0.597) and miR-19a-3p (AUC = 0.596) BC diagnosis ability is very low.

**Table 3:** Outcomes of ROC and Youden index for miR-17-92 cluster members.

	AUC	Standard Error	95% CI	Sensitivity (%)	Specificity (%)
miR-17-5p	0.845	0.030	0.782 - 0.895	84.34	71.11
miR-18a-5p	0.597	0.043	0.520 - 0.671	90.36	31.11
miR-19a-3p	0.596	0.045	0.519 - 0.670	42.17	86.67
miR-20a-5p	0.806	0.033	0.739 - 0.862	74.70	74.44
miR-19b-3p	0.741	0.037	0.669 - 0.804	72.29	66.67
miR-92a-3p	0.902	0.022	0.847 - 0.942	92.77	71.11
three-miRNA panel	0.969	0.011	0.931 - 0.989	90.36	94.44

AUC, area under curve; CI, confidence interval.

### Construction of BC Diagnostic Model

Usually, one single biomarker is unable to achieve great sensitivity and specificity simultaneously. To achieve better diagnostic ability, it is practicable to combine several miRNAs into one diagnostic model. Therefore, we selected three miRNAs (miR-17-5p, miR-20a-5p and miR-92a-3p) with AUC over 0.8 to construct a diagnostic model using the stepwise logistic regression method. We found that the AUC for the three-miRNA model was 0.969 (95% CI: 0.931 - 0.989; sensitivity = 90.36%, specificity = 94.44%; **Figure 5B**). The Hosmer-Lemeshow P value of the model was 0.885, suggesting adequate calibration. The model was calculated with the formula:


$$\text{Logit}(\text{P})=2.43\times \text{Ex}{\text{p}}_{\text{miR}-17-5\text{p}}+1.64\times \text{Ex}{\text{p}}_{\text{miR}-20\text{a}-5\text{p}}+3.51\times \text{Ex}{\text{p}}_{\text{miR}-92\text{a}-3\text{p}}-11.60$$

### External Validation of miR-17-92 Cluster Expression in Sera of BC Patients

**Figure 6:**
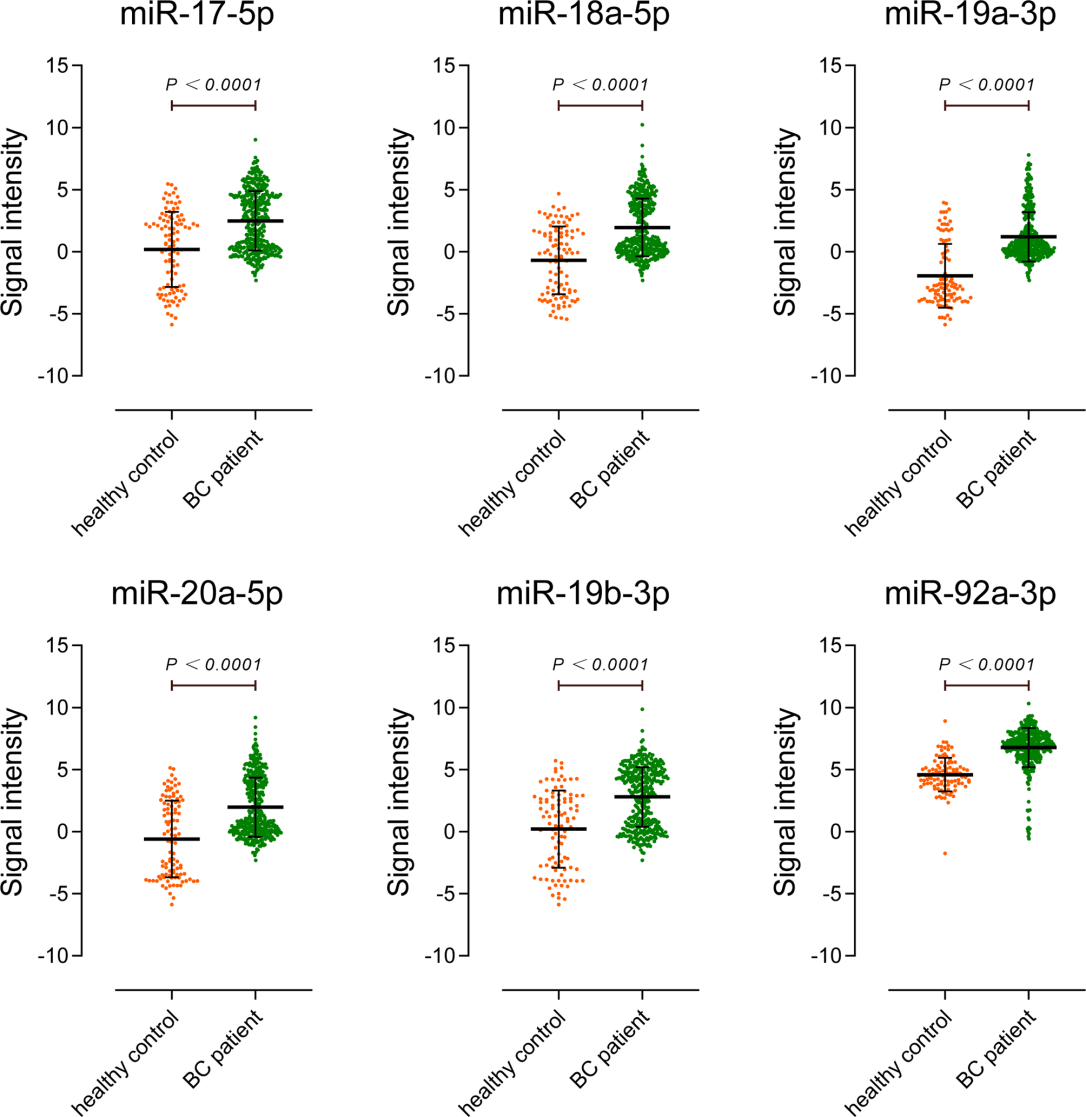
External validation of miR-17-92 cluster expression in sera of bladder cancer patients. The validation set included 392 BC patients and 100 non-cancer controls.

To further prove our results, GSE113486 was used as the external validation set for this study. GSE113486 contained 392 BC serum samples and 100 control serum samples. **Figure 6** shows the signal intensity of miR-17-92 cluster members in each group of samples. Affected by the sensitivity of gene microarray detection and the low titer of miRNA expression in serum, many cases of the miR-17-92 cluster members were undetected, both in the tumor and the control group. However, this situation had no much effect on the results. The results in **Table 2** show that the logFC values of miR-17-92 cluster members were all above 2, meaning they were significantly up-regulated in the sera of BC patients from the external validation set. But their absolute expression levels in serum are different. For example, the average expression level of miR-19a-3p is only 0.56, indicating that it is low both in the sera of patients and in the sera of controls. In contrast, the average expression level of miR-92a-3p is 6.32, indicating that it has a high basal content in serum and is more suitable as a biomarker.

### Origin of Serum miR-17-92 Cluster

To verify the hypothesis that serum miR-17-92 cluster was released into circulation by BC tumor cells, we detected the miR-17-92 cluster relative expression levels in a normal transitional epithelial cell line SV-HUC-1 and 5 BC cell lines. The results showed that miR-17-5p, miR-18a-5p, miR-20a-5p, miR-19b-3p, miR-92a-3p were significantly upregulated in three or more BC cell lines, especially in 5637, T24 and UM-UC-3 (**Supplementary Figure S1A**). miR-19a-3p was only upregulated in UM-UC-3 cell line. Later, we detect miR-17-92 cluster expression in the culture media at different time points after cultivation. We observed that expression level of the miR-17-92 cluster from culture media of 5637, UM-UC-3 and T24 cell lines increased with time while no obvious change was found in normal transitional epithelial cell line SV-HUC-1 culture media (**Supplementary Figure S1B**). These results indicated that BC-related miR-17-92 cluster could enter into the cell culture media.

### Prognostic Value of Serum miR-17-92 Cluster in Predicting Recurrence of BC Patients

To explore the prognostic value of serum miR-17-92 cluster, we followed up the BC patients after surgery to observe if their tumors recur. Among them, 8 BC patients had undergone radical cystectomy and therefore were excluded. Other patients, including 26 with TaN0M0, 30 with T1N0M0, and 10 with T2N0M0, were treated with transurethral resection of bladder tumor surgery (TURBT). After surgery, these patients were treated by bladder perfusion chemotherapy with gemcitabine. Patients were given pirarubicin at 30 mg per week for first 8 weeks after surgery, followed by pirarubicin at 30 mg per month for 10 months. In the patients we followed, all recurrences occurred in the bladder. The results of univariate Cox regression analysis showed that pathological grade, miR-18a-5p, miR-20a-5p, miR-19b-3p, and miR-92a-3p were correlated with the recurrence of BC (**Table S1**). In multivariate Cox regression analysis, miR-20a-5p and miR-92a-3p were the more significant factors (**Table S1**). The results of Kaplan-Meier analysis revealed that high serum miR-20a-5p expression was correlated with high recurrence risk in BC patients (p = 0.004) (**Figure S2**). The rest of miR-17-92 cluster members showed no significant correlation with recurrence in Kaplan-Meier analysis.

## Discussion

Liquid biopsy is a very promising method that has been extensively researched over the last decade. Liquid biopsy is anticipated to be used as the foundation for precise medical patient selection, including therapy selection and real-time monitoring of treatment impact. Furthermore, liquid biomarkers in urine and blood, such as DNA methylation and mutations, protein-based assays, gene signatures, and non-coding RNAs, might pave the way for molecular diagnosis and tailored therapy of bladder cancer (19). After therapy, biomarkers in urine may be useful in estimating residual illness or recurrence of bladder cancer; hence, liquid biopsy in urine may be a valuable source of personalized medicine prognostic biomarkers (19).

Emerging research indicates that microRNAs have tremendous promise for use in the diagnosis, prognosis, and therapy of urinary malignancies, and that microRNAs have considerable potential as biological fluid indicators of urinary system tumors, such as serum and urine (20). For example, Sebastian L Hofbauer et al. identified a 6-microRNA signature in urine for diagnosis of bladder cancer (21) and Wataru Usuba et al. identified a 7-miRNA panel in serum for specific and early detection in bladder cancer (22). In terms of prognosis research, the study found that let-7c cluster evaluation may enhance prognosis by recognizing patients’ risk of progression and addressing early radical therapy in high grade non-muscle-invasive bladder cancer (23).

Early detection of BC is also critical to a better prognosis and quality of life. Because it is non-invasive, serum-based miRNA screening is an unique and widely available diagnostic method. In this study, we selected the serum miR-17-92 cluster members (miR-17-5p, miR-18a-5p, miR-19a-3p, miR-20a-5p, miR-19b-3p and miR-92a-3p) as candidate biomarkers for BC diagnosis.

The miR-17-92 cluster is located in the open reading frame 25 of chromosome 13 (C13orf25) and is a highly conserved polycistronic miRNA cluster. The miRNA-17-92 cluster is highly expressed in various tumor cells, such as lung cancer, breast cancer, pancreatic cancer, prostate cancer and BC (24, 25). Therefore, it is also called “oncomiR1”. Recently, basic researches on miR-17-92 cluster members in BC have also received attention. For example, modification of the SNHG16/miR-17-5p/TIMP3 signal may help delay the progression of BC (15). Circ-ITCH upregulates the expression of miR-17-5p target genes p21 and PTEN by stimulating miR-17-5p, thereby inhibiting the malignant biological behavior of BC (14). miR-19a-3p promotes BC invasion and EMT by targeting RhoB (26). Similarly, miR-20a-5p can also promote BC cell growth and invasion by targeting PTEN (16).

Nonetheless, the connection between serum miR-17-92 cluster and BC has not been studied. Also, there are no studies on the clinical application of miR-17-92 cluster members in the diagnosis and treatment of BC. The present study aimed to explore the potential of miR-17-92 cluster to be diagnostic biomarkers for BC and the results were satisfactory. We confirmed the overexpression of miR-17-92 cluster in BC tissues using TCGA-BLCA data, which was consistent with previous studies. We then evaluated the differential expression of miR-17-92 cluster in serum. It was found that serum miR-92a-3p (AUC = 0.902) has extremely high BC diagnostic ability. Serum miR-17-5p (AUC = 0.845), miR-20a-5p (AUC = 0.806) and miR-19b-3p (AUC = 0.741) have moderate diagnostic ability. Then, we constructed a three-miRNA diagnostic model through logistic regression to improve specificity and sensitivity. The AUC of the model is as high as 0.969 (95% CI: 0.931 - 0.989; sensitivity = 90.36%, specificity = 94.44%), showing an excellent diagnostic ability for BC. The following external data validation (492 cases) also confirmed the reliability of our results. We also proved the overexpression of miR-17-92 cluster in BC cell lines. And we observed that miR-17-92 in the culture media of BC cell lines was gradually increased with incubation times, supporting our hypothesis that elevated serum miR-17-92 cluster was released into circulation by BC tumor cells. Among members of miR-17-92 cluster, serum miR-20a-5p expression level was associated with the recurrence of BC patients and have the potential to serve as a prognostic indicator.

In spite of the meaningful findings we obtained, certain limitations should be mentioned. All sera used in this study were taken from patients before surgery and postoperative sera were deficient. Moreover, the sample size of this study was relatively small, and multicenter study in the future is needed. The follow-up period after surgery was not long enough to obtain a more accurate conclusion. We will continue to recruit new cases and follow up, to furtherly confirm the clinical value of miR-17-92 cluster in our future work. Also, microRNAs in urine have great prospects in the diagnosis of urinary tumors (20). We will further evaluate the miRNA model in urine of patients with bladder cancer to test the role of the tool as a non-invasive biomarker for urine. Many miRNAs are involved in the inhibition of chemoresistance, while others are involved in the induction of chemoresistance (27). For example, miR-17-5p is overexpressed in pancreatic cancer and miR-17-5p inhibitor heightens the sensitivity of gemcitabine chemotherapy by up regulating the expression of Bim (28). It’s worth evaluating the expression levels of the miR-17-92 cluster correlate with the pharmacological response to drugs conventionally used in bladder treatment.

In conclusion, we confirmed that miR-17-92 cluster expression was significantly upregulated in BC tissue, cell lines, and serum compared to normal controls. Serum miR-17-92 cluster members could serve as potential diagnostic biomarkers for BC. Among them, the three-miRNA model constructed by miR-92a-3p, miR-17-5p and miR-20a-5p exhibited excellent diagnostic ability (AUC = 0.969), showing its potential to be a new noninvasive biomarker for the diagnosis of BC.

## Data Availability Statement

The datasets presented in this study can be found in online repositories. The names of the repository/repositories and accession number(s) can be found in the article/**Supplementary Material**.

## Ethics Statement

This study had been approved by the Ethics Committee of Peking University Shenzhen Hospital. The patients/participants provided their written informed consent to participate in this study.

## Author Contributions

JW, XP, YL, and ZC conceived and designed this study. JW, XP, KL, and CZ performed the experiments. XC, GH, and LZ collected the serum samples and the clinical information. JW, XP, and RL performed statistical analysis and wrote the original manuscript. YL and ZC reviewed and edited the manuscript. All authors contributed to the article and approved the submitted version.

## Funding

This study was supported by Shenzhen High-level Hospital Construction Fund, Basic Research Project of Peking University Shenzhen Hospital (JCYJ2017001, JCYJ2017004, JCYJ2017005, JCYJ2017006, JCYJ2017007, JCYJ2017012), Clinical Research Project of Peking University Shenzhen Hospital (LCYJ2017001), Science and Technology Development Fund Project of Shenzhen (no. JCYJ20180507183102747) and Clinical Research Project of Shenzhen Health Commission (no. SZLY2018023).

## Conflict of Interest

The authors declare that the research was conducted in the absence of any commercial or financial relationships that could be construed as a potential conflict of interest.

## Publisher’s Note

All claims expressed in this article are solely those of the authors and do not necessarily represent those of their affiliated organizations, or those of the publisher, the editors and the reviewers. Any product that may be evaluated in this article, or claim that may be made by its manufacturer, is not guaranteed or endorsed by the publisher.
