# Supplementary material for: Evaluation of Serum miR-17-92 Cluster as Noninvasive Biomarkers for Bladder Cancer Diagnosis
Source: Front Oncol. 2021 Dec 22;11:795837. doi: 10.3389/fonc.2021.795837 (PMC8727362; doi:10.3389/fonc.2021.795837)
Supplement: Supplementary file 1 [file DataSheet_1.docx]

Supplementary Material

## Supplementary Figures


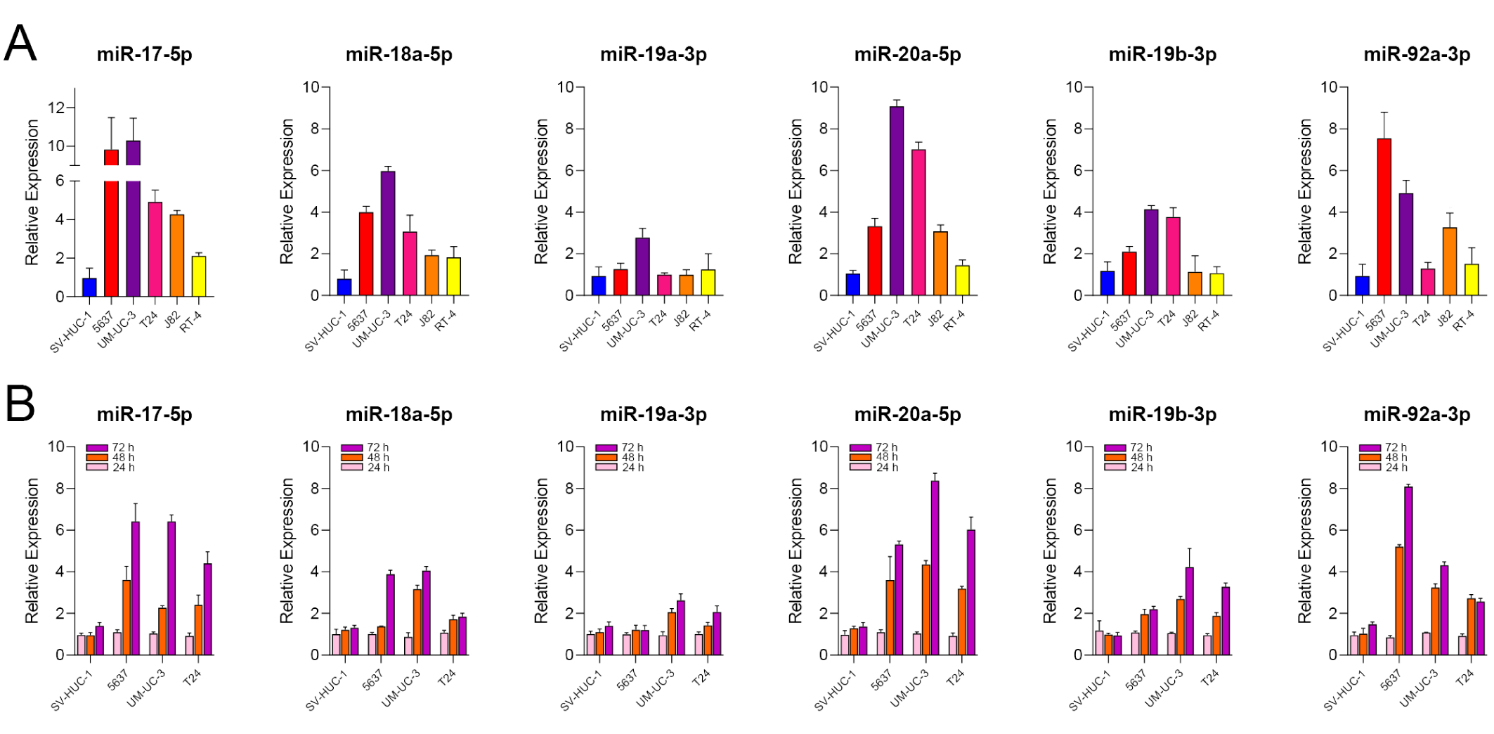


**Supplementary Figure 1.** Origin of circulating miRNAs. (a) The expression levels of miR-17-92 cluster members in the normal transitional epithelial cell (SV-HUC-1) and BC cell lines (5637, UM-UC-3, T24, J82 and RT4). (b) The expression levels of miR-17-92 cluster members in cell culture medium were measured by qRT-PCR after incubation for 24h, 48h, and 72h

**
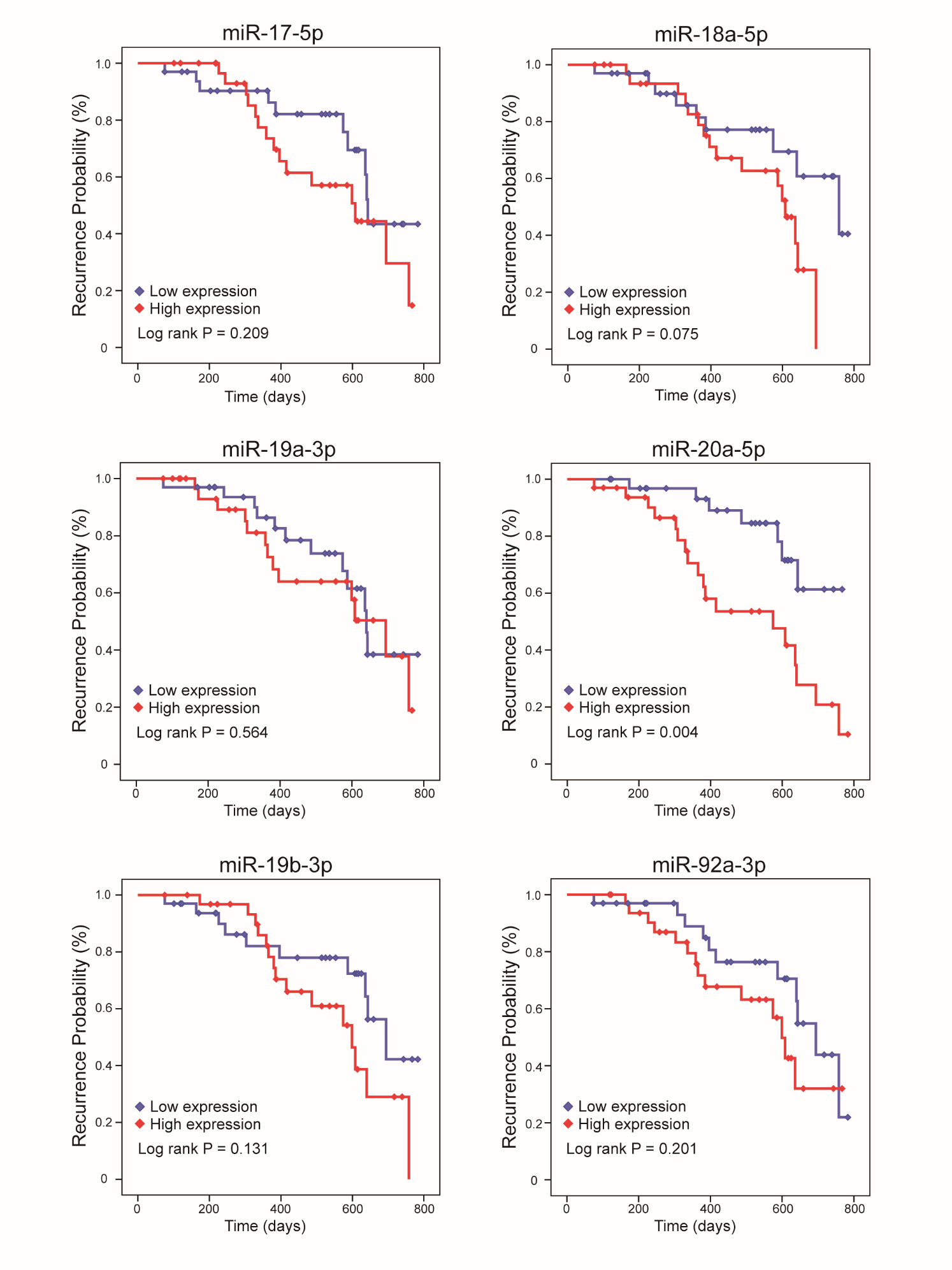
**

**Supplementary Figure 2.** Relapse-free survival curves of serum miR-17-92 cluster in bladder cancer.

## Supplementary Tables

**Supplementary Table S1** Serum miR-17-92 expression and bladder cancer recurrence

| Variable | Univariate analysis | | Multivariate analysis | |
| --- | --- | --- | --- | --- |
|  | HR(95% CI) | P-value | HR(95% CI) | P-value |
| Gender | 0.879(0.296-2.608) | 0.816 |  |  |
| Age | 0.999(0.971-1.027) | 0.917 |  |  |
| Pathological grade | 2.412(1.055-5.516) | 0.037 | 1.979(0.841-4.658) | 0.118 |
| miR-17-5p | 1.381(0.772-2.471) | 0.277 |  |  |
| miR-18a-5p | 1.658(1.030-2.670) | 0.037 | 1.519(0.919-2.509) | 0.103 |
| miR-19a-3p | 0.856(0.614-1.194) | 0.361 |  |  |
| miR-20a-5p | 2.390(1.277-4.474) | 0.006 | 1.810(0.921-3.558) | 0.085 |
| miR-19b-3p | 1.750(0.999-3.065) | 0.050 | 1.434(0.697-2.950) | 0.327 |
| miR-92a-3p | 2.467(1.089-5.586) | 0.030 | 2.224(0.956-5.176) | 0.064 |

HR, Hazard ratio; 95% CI, 95% Confidence interval.
